# Clinical presentation and treatment outcomes of gastric adenocarcinoma patients: a retrospective study from Ain Shams Clinical Oncology Department

**DOI:** 10.3332/ecancer.2025.1861

**Published:** 2025-02-27

**Authors:** Mohamed Osama Alorabi, Mohamed El-Bassiouny, Dalia Abd El Ghany El Khodary, Mai Mohamed Ali Ezz El Din, Alaa Mohamed Mohamed Ahmed Elsayed, Christine Reda

**Affiliations:** Department of Clinical Oncology and Nuclear Medicine, Faculty of Medicine, Ain Shams University, Cairo 11566, Egypt

**Keywords:** gastric cancer, Egypt, epidemiology, clinical outcomes, retrospective study

## Abstract

**Background:**

Gastric adenocarcinoma (GAC) has a different epidemiological profile in Egypt than in other countries. It ranks 11th in incidence, with 3,285 new cases and 10th in mortality, with 2,469 cases. This retrospective study aims to analyze gastric cancer epidemiology and clinical outcomes in Egyptian patients at Ain Shams University Clinical Oncology Department.

**Methods:**

We conducted a retrospective analysis of the complete medical records of patients with confirmed GAC at the Ain Shams University Clinical Oncology Department from January 2017 to December 2020.

**Results:**

This study included 70 patients with GAC. The median age was 52.5 years, with nearly half of cases under 50 years and males representing 53% of the cohort. 70% of patients were from urban areas. Nearly one-third were smokers, with 57.1% having medical comorbidities, mainly diabetes mellitus, hypertension and viral hepatitis. Additionally, 25.7% had a positive family history of GAC. Most Common presenting symptoms were vomiting (42.9%) and abdominal pain (57.1%). 40% of tumours were in the gastric body, and 64.3% were diffuse-type GAC, with 64.3% classified as high grade (III). At presentation, the majority of cases were metastatic (55.7%), with 15.7% presenting with stage II disease and 28.6% with stage III. Most patients (72.8%) had an Eastern Cooperative Oncology Group ≤2. Only 18.6% received neoadjuvant chemotherapy, while 48.6% underwent surgical resection with adequate lymph node dissection in 55.9% of cases. Adjuvant chemotherapy or chemoradiation was administered to 19 patients. The median overall survival (OS) was 11 months, 36 months for stage II, 17 months for stage III and 7 months for stage IV. Univariate analysis indicated that female gender, higher stage (Stage III-IV), higher grade (G IV), absence of neoadjuvant chemotherapy and intestinal type were significantly associated with increased mortality. However, multivariate analysis adjusting for these factors identified the advanced stage as a significant independent predictor of mortality.

**Conclusion:**

This study identified the distinct GAC profile of Egyptian patients, younger age, aggressive tumours and frequent metastases. These factors contributed to lower OS. Further research and targeted interventions are needed to improve outcomes.

## Introduction

Gastric adenocarcinoma (GAC) was the fifth cancer in incidence most common cancer worldwide, with 968,350 newly diagnosed cancer cases in 2020. Additionally, it was the fifth in cancer-related mortality, with 659,853 reported cases [1]. Countries in East Asia and East Europe have the highest incidence rates, while Northern America and Northern Europe have rates similar to those in Africa, which are generally low. This geographical variation reflects epidemiological differences between countries [1, 2]. Recent studies in the Middle East and North Africa (MENA) region reveal significant variability in GAC incidence and associated risk factors. For instance, the incidence in Egypt remains one of the lowest in the region at 3.5 per 100,000. At the same time, Iran reports significantly higher rates at 14.6 per 100,000, reflecting differences in genetic, dietary and environmental risk factors [3]. Dietary patterns, including high salt consumption and low fruit and vegetable intake, are critical in shaping gastric cancer risk across the region [4]. Despite lower overall incidence compared to global averages, MENA countries report higher mortality-to-incidence ratios, underscoring challenges in early detection and treatment accessibility [5]. Addressing these disparities through region-specific cancer control strategies, including early screening and targeted prevention efforts, remains an urgent priority [4, 5].

There are two main histologic subtypes of GAC: intestinal and diffuse. These subtypes differ in prevalence, predisposing factors, pathogenesis and management [6]. Another classification is based on topography. It identifies two distinct epidemiological entities: the cardia, which refers to the upper stomach, and the non-cardia, which refers to the lower stomach [7, 8]. In addition to histological and topographical classifications, the Borrmann [9] classification is frequently utilised to describe GAC based on their macroscopic appearance. It divides tumours into four categories: polypoid carcinoma (Type I), fungating carcinoma (Type II), ulcerated carcinoma (Type III) and diffusely infiltrative carcinoma (Type IV). This system aids in correlating morphological features with disease prognosis [10, 11].

*Helicobacter pylori* (H. pylori) induced gastritis is a recognised predisposing factor for GAC [12]. Furthermore, variables such as family history, smoking, alcohol consumption and dietary habits, specifically the excessive intake of salt-cured foods, processed red meat, poultry or fish and low fruit consumption, contribute to the chance of developing GAC [12, 13].

Early GAC cases can be asymptomatic; however, in advanced stages, disease progression often leads to significant weight loss, dysphagia, epigastric or vague abdominal pain, vomiting and even severe upper gastrointestinal tract bleeding [14].

Gastric carcinomas are typically detected in an advanced stage, significantly impacting the available treatment choices [15]. In general, gastric carcinoma can be managed with surgical resection along with neoadjuvant/adjuvant chemotherapy and may or may not require radiotherapy, depending on the stage [16]. However, patients with distant metastases have a poor prognosis, with less than 1 year of median overall survival (OS) [17].

Advancements in understanding molecular markers and immunotherapy have significantly improved GAC management. Human epidermal growth factor receptor 2 (HER2) is a crucial marker overexpressed in 15%–20% of GC cases, predominantly in intestinal-type adenocarcinomas [18]. The landmark ToGA trial established trastuzumab, a monoclonal antibody targeting HER2, as a standard treatment for HER2-positive advanced GC [19], significantly improving OS when combined with chemotherapy [18]. Immunotherapy has also emerged as a promising approach, particularly immune checkpoint inhibitors (ICIs) such as nivolumab and pembrolizumab, which target PD-1/PD-L1 pathways. These agents show remarkable efficacy in patients with microsatellite instability-high or Epstein-Barr virus-positive tumours. As recommended by recent trials, combining ICIs with HER2-targeted therapies and chemotherapy has further enhanced outcomes for advanced and metastatic cases [20].

In Egypt, GAC presents a distinct epidemiological profile compared to other countries. According to Globocan [21], it ranks as the 11th most common cancer, with 3,285 new cases and the 10th leading cause of cancer-related mortality, accounting for 2,469 deaths. Despite this burden, data on the epidemiology and clinical characteristics of GAC in Egypt remain limited, highlighting a significant knowledge gap. Addressing this gap is essential for a comprehensive understanding of the disease within the country.

This study aims to analyze the epidemiology, clinicopathological features and treatment outcomes of GAC among Egyptian patients.

## Methods

### Study setting

This retrospective cohort study was conducted at the Department of Clinical Oncology, Ain Shams University teaching hospitals, and included gastric carcinoma patients treated between January 2017 and December 2020.

### Eligibility criteria

Adult patients aged 18 years or older with a confirmed diagnosis of primary GAC, verified through endoscopic biopsy or surgical pathology, were included. Patients were eligible if complete medical records were available for the study period. Exclusion criteria encompassed cases of non-adenocarcinoma gastric pathologies and diagnoses of secondary malignancies.

### Ethical considerations

The study adhered to the ethical principles outlined in the Declaration of Helsinki and received approval from the Institutional Review Board of Ain Shams Faculty of Medicine (IRB-FMASU MS 299/2022).

### Data collection

Data were extracted from patient medical records, capturing demographic characteristics (age, gender), clinical performance status (based on the Eastern Cooperative Oncology Group (ECOG) score) [22], comorbidities, personal habits, endoscopic findings, tumour characteristics (size, location, histopathology), treatment interventions (surgical and perioperative therapies), metastatic treatments, treatment responses and survival outcomes. Tumour response was assessed using the Response Evaluation Criteria in Solid Tumours version 1.1 [23], and staging was classified according to the American Joint Committee on Cancer TNM 8th edition guidelines [24].

Efforts were made to minimise bias through consistent data collection procedures and validation of clinical records. To maintain confidentiality, all data were anonymised by removing identifiable patient information and securely storing the dataset, accessible only to authorised personnel.

### Statistical analysis

The statistical analysis was conducted using IBM SPSS Statistics software (Version 26.0, IBM Corporation, Armonk, NY, USA). Data were first coded and organised in Microsoft Excel 2016 before being imported into SPSS for analysis. The Kolmogorov-Smirnov test was used to assess the normality of data distribution. Continuous variables with normal distribution were expressed as mean ± standard deviation, while those with non-normal distribution were presented as median and interquartile range. Categorical variables were summarised as frequencies and percentages.

Survival analysis was performed to evaluate OS and progression-free survival using the Kaplan–Meier method. Differences between survival curves were tested using the log-rank test to determine statistical significance. The Cox proportional hazards regression model was utilised for univariate and multivariate analyses to identify factors associated with survival outcomes. Results from the Cox regression were reported as hazard ratios with 95% confidence intervals (CIs).

To account for potential confounding factors, multivariate analysis included variables that were statistically significant in univariate analysis or deemed clinically relevant. Subgroup analyses were conducted to explore the impact of key demographic and clinicopathological variables on survival outcomes. All statistical tests were two-tailed and a *p*-value <0.05 was considered indicative of statistical significance.

## Results

### Patient demographics and clinical characteristics

The characteristics of the 70 studied patients with GACare summarised in Table 1. The median age was 52.2 years, ranging from 22.0 to 86.0 years. Age distribution revealed that 47.1% of patients were younger than 50, while 52.9% were aged 50 years or older. Gender distribution was nearly equal, with 52.9% male and 47.1% female patients. Performance status assessment revealed a varied distribution, with 37.1% categorised as Grade I, 35.7% as Grade II, 18.6% as Grade III and 8.6% as Grade IV. Most patients resided in urban areas (70.0%). Alcohol consumption was reported in only 1.4% of patients, while the majority (98.6%) reported no alcohol intake. Similarly, 67.1% were non-smokers, 25.7% were current smokers and 7.1% were ex-smokers. Over half of the patients (57.1%) had medical comorbidities, with diabetes mellitus (37.5%) and hypertension (32.5%) being the most prevalent. Family history of gastric cancer was positive in 25.7% of cases. *Helicobacter pylori* infection was found in 15.7% of GAC patients, while 84.3% were free of the infection. Symptoms and signs commonly observed included abdominal pain (57.1%), vomiting (42.9%) and weight loss (21.4%). Most tumours were in the gastric body (40.0%), followed by the antrum (17.1%) and fundus (15.7%). Most tumours exhibited a fungating mass (62.9%) on imaging. Pathological analysis revealed that most tumours were of the diffuse type (64.3%). Grading indicated that most tumours were Grade IV (64.3%). Tumour markers CEA and CA 19.9 were elevated in 11.4% and 12.9% of patients, respectively, while the majority had unknown marker levels (CEA: 64.3%, CA 19.9: 68.5%). Most patients were diagnosed at advanced stages, with 55.7% at Stage IV. The most common sites of metastasis are the peritoneum (53.8%), ascites (46.2%) and liver (23.1%).

### Treatment patterns and chemotherapy regimens

**Figure 1.**
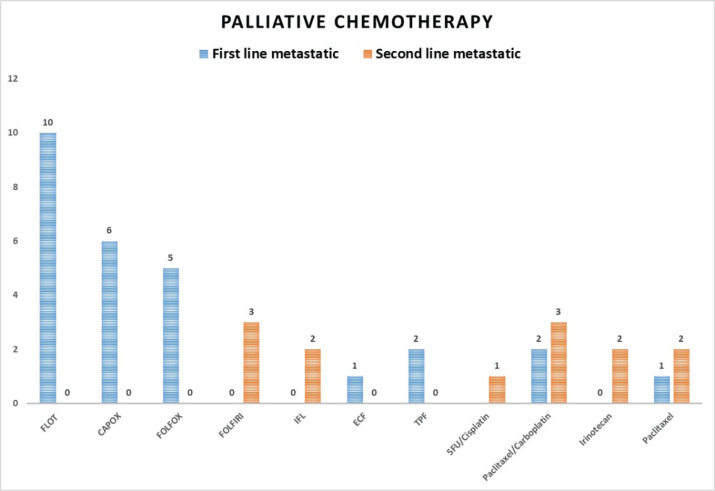
Palliative chemotherapy regimens in GAC.

In the treatment analysis outlined in Table 2, approximately half of the patients (48.6%) underwent surgical intervention, with total gastrectomy performed in 32.4% of cases, sub-total gastrectomy in 58.8% and palliative gastro-jejunostomy in 8.8%. Lymph node dissection was carried out in 88.2% of surgical cases, of which 55.9% received adequate lymph node dissection. Neoadjuvant chemotherapy was administered to 18.6% of patients, with various regimens utilised, including FLOT (Docetaxel, oxaliplatin, leucovorin and 5-fluorouracil) in 61.5% of cases, FOLFOX (Oxaliplatin, leucovorin and 5-fluorouracil) in 15.4% and others such as DOX (Docetaxel and oxaliplatin), ECX (Epirubicin, cisplatin and capecitabine), and GEMOX (Gemcitabine and oxaliplatin). Adjuvant therapy was administered in 27.1% of patients, with treatment protocols including the Macdonald protocol, FLOT followed by concurrent chemoradiation with Capecitabine, CapeOX followed by radiotherapy and others such as DOX and TPF (Docetaxel, cisplatin and 5-fluorouracil). Palliative chemotherapy, initiated at the start or upon disease progression, was given to 40.0% of patients, with specific regimens tailored to individual patient needs and disease progression. Figure 1 depicts the palliative chemotherapy protocols utilised in this study, illustrating the treatment sequences administered to manage GAC progression. In the first-line chemotherapy category, various protocols were employed, including FLOT for ten patients, CapeOX for six patients, FOLFOX for five patients and other regimens such as Paclitaxel/Carboplatin, TPF, Weekly Paclitaxel and ECF. Moving to the second-line chemotherapy, diverse treatment strategies were initiated based on individual patient responses, with regimens including, Paclitaxel/Carboplatin, Weekly Paclitaxel, FOLFIRI, IFL, 5-FU/Cisplatin and Irinotecan being administered. The third and fourth-line chemotherapy also demonstrated variability, with treatments such as Paclitaxel/Carboplatin, FOLFIRI, Irinotecan and Capecitabine being administered in response to disease progression.

### Survival analysis

**Figure 2.**
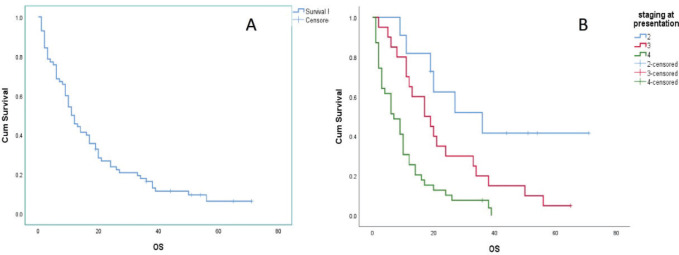
OS analysis of GAC patients: (a): Across the entire study population and (b): Stratified by disease stage.

The survival data, illustrated in Figure 2, provides crucial insights into the prognosis of GAC patients at various disease stages. Patients diagnosed with Stage II disease had a significantly longer median OS of 36.0 months (95% CI: 12.429–59.571). However, as the disease advanced to Stage III and IV, median OS times markedly declined to 17.0 months (95% CI: 7.139–26.861) and 7.0 months (95% CI: 3.176–10.824), respectively. Considering all stages collectively, the median OS was 11.0 months (95% CI: 8.018–13.982). The comparison of OS curves using the Log-rank test showed a significant difference (Chi-squared = 20.51, DF = 2, Significance *p* < 0.001).

In Table 3, the analysis of various parameters revealed significant associations with mortality in GAC patients. In the univariate analysis, female gender (*p* = 0.036; OR = 9.846; 95% CI: 1.156–83.879), higher grade (Grade IV) tumours (*p* = 0.023; OR = 2.537; 95% CI: 1.137–5.659) and the diffuse histopathological type (*p* = 0.026; OR = 8.361; 95% CI: 1.582–44.195) were significantly associated with increased mortality risk. Additionally, the absence of neoadjuvant chemotherapy (*p* = 0.020; OR = 0.176; 95% CI: 0.041–0.764) and advanced stage (Stage III-IV) (*p* = 0.002; OR = 5.978; 95% CI: 1.888–18.928) were also significantly associated with higher mortality rates. Furthermore, upon conducting multivariate analysis and adjusting for these factors, advanced stage (Stage III/IV) disease remained a significant independent predictor of mortality (*p* = 0.028; OR = 14.429; 95% CI: 1.34–155.0), reinforcing its importance as a prognostic indicator in GACs.

## Discussion

Gastric cancer poses a significant global burden, with poor survival rates largely attributed to late-stage diagnosis and limited treatment access. In Egypt, our findings highlight distinct epidemiological trends, including a younger median age, a high incidence of aggressive diffuse-type adenocarcinoma and a predominance of advanced-stage disease at diagnosis. These patterns likely result from insufficient screening programs, low public awareness and underutilisation of neoadjuvant therapies.

The median age in our study (52.2 years) contrasts with older mean ages reported in studies from Lebanon (72 years) [25], Spain (67.9 years) [26] and the United States [27], where most patients are over 65 years old [27]. Nearly half of our patients were younger than 50, differing from a 1979 study at NCI, Cairo, which reported a mean age of 57.49 years [28]. Age groups (<50 and ≥50 years) showed no significant association with mortality risk, consistent with several studies [25, 29, 30], although other studies suggest older age as a significant prognostic factor [31–33].

Male predominance (53%) aligns with global trends in the Globocan 2022 data, which reported higher incidence rates in males (627,229 cases) compared to females (341,121 cases) [34]. Interestingly, the observed association between female gender and lower survival in our study contrasts with findings from large-scale studies, including those from the SEER database in the United States [35], Finland [36] and a meta-analysis on sex disparity in GAC patients [37].

Survival differences in gastric cancer by age and gender are influenced by tumour biology, with younger patients more likely to have aggressive diffuse-type cancers and older adults presenting with intestinal-type cancers [35]. Estrogen’s protective effects and genetic factors may explain gender disparities [38]. Socioeconomic factors, healthcare access and lifestyle differences like smoking and *H. pylori* infection further impact outcomes [2, 35, 39]. Improved screening and tailored treatments are key to addressing these disparities.

Between 2004 and 2011, the United States reported 29,577 cases of GAC, with rural residents (10.6%) showing higher mortality due to barriers such as limited access to care and transportation challenges [40]. However, a Canadian study from 2010 to 2018 found no survival differences between urban and rural patients, likely due to consistent treatment protocols [41]. Our study reflected similar findings, which can likely be attributed to its single-centre design, standardised publicly funded healthcare system, uniform treatment protocols and the consistent involvement of the same treatment teams for all patients, irrespective of their residence.

Alcohol consumption is recognised as a risk factor for gastric cancer [42]. While some studies suggest that alcohol intake at diagnosis may reduce survival rates in gastric cancer patients [43, 44], conflicting results have been reported in other studies [45, 46]. In our study, the impact of alcohol on survival remains uncertain as only one patient reported alcohol intake. Despite smoking being a known risk factor for gastric cancer and associated with decreased survival in many cases [47, 48], our study found no impact of smoking on survival outcomes. This can be attributed to patients of our study having a relatively low number of smokers.

A family history of GAC, reported in 26% of patients, aligns with previous studies identifying it as a significant risk factor [49, 50]. For instance, a Japanese study noted a 2.15-fold higher risk in individuals with affected relatives [51].

*Helicobacter pylori* infection is a significant risk factor for gastric cancer, contributing to approximately 20% of cases globally. In this study, 15.7% of cases were associated with *H. pylori* infection. In contrast, recent research from China reports a much higher association, exceeding 60% [52]. Chinese studies have shown conflicting results on the impact of *H. pylori* on survival: one suggests a favourable outcome [53], while another found no correlation [54], consistent with the findings of this study.

Comorbidities are commonly associated with reduced survival in GAC patients [55, 56]. Our study, with a comorbidity prevalence of 57.1%, did not observe this trend. Diabetes mellitus, hypertension and viral hepatitis were the most prevalent comorbidities, but they were well-managed without major organ dysfunction, likely due to comprehensive care at our centre.

In our cohort, GACs were classified predominantly as a diffuse subtype (64.3%), associated with shorter OS, followed by the intestinal subtype (35.7%). A Taiwanese study reported a higher prevalence of intestinal-type tumours (46.3%) and fewer diffuse-type tumours (32.6%) [59]. Despite these differences, both studies highlighted the consistent prognostic value of Laurén classification across populations, aligning with findings from a German study on locally advanced gastric or gastroesophageal cancers treated with neoadjuvant/perioperative chemotherapy [60].

The distribution of tumour grades in our cohort parallels patterns observed in Arab countries such as Saudi Arabia and Lebanon, where Grade IV tumours were most common (64.3%) [25, 61]. Comparatively, Indian data [58] showed a higher proportion of poorly differentiated tumours (44.3%) and fewer well-differentiated ones (19.6%), while Chinese findings also reflected varied grade distributions [62]. These differences likely result from demographic, genetic and healthcare disparities. Grade IV tumours, recognised for their aggressive nature, were linked to poor survival, consistent with other studies [61, 63].

Advanced-stage diagnosis was predominant, with Stage IV being the most common. Peritoneal metastasis and ascites were frequent, followed by liver, lung and bone involvement. These patterns mirror trends observed in other Arab countries [25, 61], underscoring the challenges of late-stage detection due to limited awareness and inadequate screening programs. This highlights the critical need for early detection initiatives to improve outcomes for GAC patients.

The treatment patterns in this study align with guideline recommendations. For locally advanced GAC, radical gastrectomy with D2 lymph node dissection remains the standard, with guidelines recommending sampling at least 15 lymph nodes for survival benefit [24, 64]. Neoadjuvant chemotherapy, predominantly the FLOT protocol in our cohort, effectively downstaged tumours and improved curative resection rates [65]. For patients who did not receive neoadjuvant therapy and were at high recurrence risk, adjuvant chemoradiation or capecitabine plus oxaliplatin, as per the CLASSIC trial, were appropriate [66, 67]. In the first line of treatment, the most common chemotherapy regimens were the triplet combination of docetaxel, fluorouracil and a platinum compound, as well as the doublet combination of fluorouracil and a platinum compound. For second and later-line treatments, fluoropyrimidine, paclitaxel or irinotecan were frequently prescribed [64].

The disease is associated with poor survival outcomes, particularly in advanced stages. In our study, most patients presented with stage III-IV disease, with a median OS of 11.0 months. This is consistent with findings from Iran, where the median OS was reported as 16.33 months [68], and Turkey, where it was 18 months [69]. Data from the SEER database further illustrate the stage-dependent survival disparities, with median survival rates of 96 months for stage I, 30 months for stage II, 20 months for stage III and 14 months for stage IV [70]. In the multivariate analysis, only stage (III/IV) remained statistically significant, indicating it was a critical independent predictor. This underscores the importance of early detection, as advanced stages are strongly associated with worse outcomes [71–75].

The present research provides valuable insights into the epidemiological, clinicopathological characteristics and treatment outcomes of GAC patients in Egypt. The analysis of survival outcomes and treatment patterns adds to the growing body of knowledge needed to improve clinical management in the region. However, this study has some limitations. Its retrospective design may introduce biases related to data completeness and interpretation. The single-center setting and relatively small sample size may restrict the applicability of the results to other populations. Furthermore, the absence of molecular and genetic profiling limits the exploration of potential predictive or prognostic biomarkers. Future research with multicenter collaborations and molecular studies is encouraged to confirm and build upon these findings.

## Conclusion

This study highlights the younger median age and high prevalence of advanced-stage, diffuse-type GAC among Egyptian patients at Ain Shams University. The common presentation with late-stage disease reveals the urgent need for more efforts regarding early detection strategies, public awareness and optimised treatment strategies to improve outcomes for gastric cancer patients in Egypt.

## Conflicts of interest

The authors declare that there are no conflicts of interest.

## Funding

This research did not receive specific grants from public, commercial or not-for-profit funding agencies.

## Figures and Tables

**Table 1.** Patient demographics and clinical characteristics data.

Characteristic		Studied patients (*N* = 70)	
		Number	%
Age (years)	Median	52.2	
	Range	22.0–86.0	
Age distribution	< 50 years	33	47.1%
	≥ 50 years	37	52.9%
Gender	Male	37	52.9%
	Female	33	47.1%
ECOG performance status	I	26	37.1%
	II	25	35.7%
	III	13	18.6%
	IV	6	8.6%
Residence	Rural	21	30.0%
	Urban	49	70.0%
Alcohol consumption	No	69	98.6%
	Yes	1	1.4%
Smoking	Non-smoker	47	67.1%
	Current smoker	18	25.7%
	Ex-smoker	5	7.1%
Medical comorbidities	No	30	42.9%
	Yes	40	57.1%
	Diabetes mellites (DM)	15	37.5%
	Hypertension (HTN)	13	32.5%
	HCV/HBV	11	27.5%
	Cardiac	5	12.5 %
	Renal	1	2.50%
	Other	10	25.0%
Family history	Negative	52	74.3%
	Positive	18	25.7%
*H. pylori* infection	Present	11	15.7%
	Absent/Unknown	59	84.3%
Symptoms and signs	Abdominal pain	40	57.1%
	Vomiting	30	42.9%
	Hematemesis	15	21.4%
	Weight loss	15	21.4%
	Melena	14	20.0%
	Anemia	12￼	17.1%
	Dysphagia	7	10.0%
	Dyspepsia	5	7.1%
Tumor location	Cardia	6	8.6%
	Fundus	11	15.7%
	Body	28	40.0%
	Antrum	12	17.1%
	Prepyloric region	11	15.7%
	Pylorus	2	2.9%
Tumor shape	Fungating mass	44	62.9%
	Malignant ulcer	14	20.0%
	Mural thickening	12	17.1%
Pathology	Diffuse type	45	64.3%
	Intestinal type	25	35.7%
Grade	II	16	22.9%
	III	9	12.9%
	IV	45	64.3%
Tumor marker CEA	Normal	17	24.3%
	Elevated	8	11.4%
	Unknown	45	64.3%
Tumor marker CA 19.9	Normal	13	18.6%
	Elevated	9	12.9%
	Unknown	46	68.5%
Stage	I	0	0.00%
	II	11	15.7%
	III	20	28.6%
	IV	39	55.7%
Sites of metastasis	Peritoneum	21	53.8%
	Ascites	18	46.2%
	Liver	9	23.1%
	Adnexal	7	17.9%
	Lung	5	12.8%
	Pleural effusion	4	10.3%
	Bone	3	7.7%
	Splenic	3	7.7%

**Table 2.** Treatment characteristics of study participants.

Treatment modality	Studied patients (*N* = 70)	
	Number	%
Surgery for 34 cases (48.6%)		
Total gastrectomy	11	32.4%
Sub-total gastrectomy	19	58.8%
Palliative gastro-juejnostomy	3	8.8%
Lymph node dissection	30	88.2%
Adequate lymph node dissection	19	55.9%
Inadequate lymph node dissection	11	32.4%
Neoadjuvant chemotherapy for 13 cases (18.6 %)		
FLOT (Docetaxel, oxaliplatin, leucovorin, and 5-fluorouracil)	8	61.5%
FOLFOX (Oxaliplatin, leucovorin, and 5-fluorouracil)	2	15.4%
DOX (Docetaxel, and oxaliplatin)	1	7.7%
ECX (Epirubicin, cisplatin, and capecitabine)	1	7.7%
GEMOX (Gemcitabine, and oxaliplatin)	1	7.7%
Adjuvant therapy for 19 cases (27.1%)		
Macdonald protocol (postoperative combination of fluorouracil plus leucovorin and locoregional radiation therapy)	10	52.6%
FLOT (Docetaxel, oxaliplatin, leucovorin, and 5-fluorouracil) followed by concurrent chemoradiation with capecitabine	5	26.3%
CapeOX (Capecitabine, and oxaliplatin) followed by radiotherapy	2	10.5%
DOX (Docetaxel, and oxaliplatin)	1	5.3%
TPF (Docetaxel, cisplatin, and 5-fluorouracil)	1	5.3%

Palliative chemotherapy (from the start or upon progression) for 28 cases (40.0%)

**Table 3.** Logistic regression analysis for factors predicting mortality and decrease OS.

Parameters	Univariate				Multivariate			
	*p*-value	Odds ratio (OR)	95% CI		*p*-value	Odds ratio (OR)	95% CI	
			Lower limit	Upper limit			Lower limit	Upper limit
Age	0.112	3.889	0.727	20.808				
Gender (Female)	**0.036**	9.846	1.156	83.879	0.196	6.306	0.387	102.745
Smoking	0.432	1.768	0.427	7.331				
Comorbidities	0.429	1.812	0.415	7.916				
Family history	0.304	0.324	0.038	2.785				
Grade (Grade IV)	**0.023**	2.537	1.137	5.659	0.313	0.186	0.007	4.865
*H pylori*	0.687	1.569	0.176	13.968				
P.S at presentation	0.195	0.511	0.185	1.411				
Histopathological type (Diffuse)	**0.026**	8.361	1.582	44.195	0.080	8.438	0.915	77.819
Lymph node dissection	0.611	0.568	0.064	5.021				
Neoadjuvant chemotherapy	**0.020**	0.176	0.041	0.764	0.246	0.229	0.019	2.756
Adjuvant chemotherapy	0.55	0.825	0.44	1.549				
Palliative chemotherapy	0.725	0.851	0.348	2.085				
Site	0.574	0.483	0.038	6.111				
Stage (III/IV)	**0.002**	5.978	1.888	18.928	**0.028**	14.429	1.34	155.0
Surgery	0.159	0.321	0.066	1.561				

## References

[1] Sung H, Ferlay J, Siegel RL (2021). Global cancer statistics 2020: GLOBOCAN estimates of incidence and mortality worldwide for 36 cancers in 185 countries. CA Cancer J Clin.

[2] Ilic M, Ilic I (2022). Epidemiology of stomach cancer. World J Gastroenterol.

[3] Ramazani Y, Mardani E, Najafi F (2021). Epidemiology of gastric cancer in North Africa and the Middle East from 1990 to. J Gastrointest Cancer.

[4] Morgan E, Arnold M, Camargo MC (2022). The current and future incidence and mortality of gastric cancer in 185 countries, 2020–40: a population-based modelling study. EClinicalMedicine.

[5] Al-Muftah M, Al-Ejeh F (2023). Cancer incidence and mortality estimates in Arab countries in 2018: a GLOBOCAN data analysis. Cancer Epidemiol Biomarkers Prev.

[6] Ajani JA, Lee J, Sano T (2017). Gastric adenocarcinoma. Nat Rev Dis Primers.

[7] de Martel C, Georges D, Bray F (2020). Global burden of cancer attributable to infections in 2018: a worldwide incidence analysis. Lancet Glob Health.

[8] Thrift AP, Wenker TN, El-Serag HB (2023). Global burden of gastric cancer: epidemiological trends, risk factors, screening and prevention. Nat Rev Clin Oncol.

[9] Borrmann R, Henke F, Lubarsch O (1926). Geschwülste des Magens und Duodenum. Handbuch der speziellen pathologischen Anatomie und Histologie. IV/1.

[10] Díaz Del Arco C, Ortega Medina L, Estrada Muñoz L (2021). Are Borrmann's types of advanced gastric cancer distinct clinicopathological and molecular entities? A western study. Cancers.

[11] Song XH, Zhang WH, Chen XL (2020). Prognostic impact of Borrmann classification on advanced gastric cancer: a retrospective cohort from a single institution in western China. World J Surg Oncol.

[12] Kesharwani A, Dighe OR, Lamture Y (2023). Role of helicobacter pylori in gastric carcinoma: a review. Cureus.

[13] Zali H, Rezaei-Tavirani M, Azodi M (2011). Gastric cancer: prevention, risk factors and treatment. Gastroenterol Hepatol Bed Bench.

[14] Marghalani AM, Salman TOB, Faqeeh FJ (2020). Gastric carcinoma: insights into risk factors, methods of diagnosis, possible lines of management, and the role of primary care. J Fam Med Prim Care.

[15] Liu Y, Chen L, Zhang R (2020). Efficacy and safety of elemene combined with chemotherapy in advanced gastric cancer: a meta-analysis. Medicine.

[16] Joharatnam-Hogan N, Shiu KK (2020). Challenges in the treatment of gastric cancer in the older patient. Cancer Treat Rev.

[17] Carcas LP (2014). Gastric cancer review. J Carcinog.

[18] Sato Y, Okamoto K, Kawano Y (2023). Novel biomarkers of gastric cancer: current research and future perspectives. J Clin Med.

[19] Bang YJ, Van Cutsem E, Feyereislova A (2010). Trastuzumab in combination with chemotherapy versus chemotherapy alone for treatment of HER2-positive advanced gastric or gastro-oesophageal junction cancer (ToGA): a phase 3, open-label, randomised controlled trial. Lancet.

[20] Triantafillidis JK, Konstadoulakis MM, Papalois AE (2024). Immunotherapy of gastric cancer: present status and future perspectives. World J Gastroenterol.

[21] Ferlay J EM, Lam F, Laversanne M (2024). https://gco.iarc.who.int/media/globocan/factsheets/populations/818-egypt-fact-sheet.pdf.

[22] Zubrod CG, Schneiderman M, Frei III E (1960). Appraisal of methods for the study of chemotherapy of cancer in man: comparative therapeutic trial of nitrogen mustard and triethylene thiophosphoramide. J Chronic Dis.

[23] Eisenhauer EA, Therasse P, Bogaerts J (2009). New response evaluation criteria in solid tumours: revised RECIST guideline (version 1.1). Eur J Cancer.

[24] Amin MB, Edge SB, Greene FL (2017). AJCC Cancer Staging Manual.

[25] El Halabi M, Horanieh R, Tamim H (2020). The impact of age on prognosis in patients with gastric cancer: experience in a tertiary care centre. J Gastrointest Oncol.

[26] Díaz del Arco C, Ortega Medina L, Estrada Muñoz L (2023). Impact of age at diagnosis on clinicopathological features, prognosis, and management of gastric cancer: a retrospective single-center experience from Spain. Cancers.

[27] Wang SJ, Emery R, Fuller CD (2007). Conditional survival in gastric cancer: a SEER database analysis. Gastric Cancer.

[28] Omar S, Ibrahim AS, El-Aaser AA (1979). A study of gastric cancer in Egypt. Jpn J Clin Oncol.

[29] Theuer CP, Virgilio C, Keese G (1996). Gastric adenocarcinoma in patients 40 years of age or younge. Am J Surg.

[30] Park JC, Lee YC, Kim JH (2009). Clinicopathological aspects and prognostic value with respect to age: an analysis of 3,362 consecutive gastric cancer patients. J Surg Oncol.

[31] Lai JF, Kim S, Li C (2008). Clinicopathologic characteristics and prognosis for young gastric adenocarcinoma patients after curative resection. Ann Surg Oncol.

[32] Yang D, Hendifar A, Lenz C (2011). Survival of metastatic gastric cancer: significance of age, sex and race/ethnicity. J Gastrointest Oncol.

[33] Li X, Wang W, Ruan C (2017). Age-specific impact on the survival of gastric cancer patients with distant metastasis: an analysis of SEER database. Oncotarget.

[34] Bray F, Laversanne M, Sung H (2024). Global cancer statistics 2022: GLOBOCAN estimates of incidence and mortality worldwide for 36 cancers in 185 countries. CA Cancer J Clin.

[35] Li H, Wei Z, Wang C (2020). Gender differences in gastric cancer survival: 99,922 cases based on the SEER database. J Gastrointest Surg.

[36] Maharjan U, Kauppila JH (2022). Gastric cancer completeness in Finnish Cancer Registry and Finnish Patient Registry: a population-based nationwide retrospective cohort study. BMJ Open.

[37] Luan X, Niu P, Wang W (2022). Sex disparity in patients with gastric cancer: a systematic review and meta-analysis. J Oncol.

[38] Gan X, Dai G, Li Y (2024). Intricate roles of estrogen and estrogen receptors in digestive system cancers: a systematic review. Cancer Biol Med.

[39] Kalff MC, Wagner AD, Verhoeven RH (2022). Sex differences in tumor characteristics, treatment, and outcomes of gastric and esophageal cancer surgery: nationwide cohort data from the Dutch Upper GI Cancer Audit. Gastric Cancer.

[40] Minhas AA, Fatima Z, Kommineni SK (2021). The association of rural-urban inhabitation with gastric adenocarcinoma mortality and treatment: a surveillance, epidemiology, and end results (seer)-based study. Cureus.

[41] Kammili A, Morency D, Cools-Lartigue J (2023). Remoteness from urban centre does not affect gastric cancer outcomes with established care pathway to specialist centre. Can J Surg.

[42] Li Y, Eshak ES, Shirai K (2021). Alcohol consumption and risk of gastric cancer: the Japan collaborative cohort study. J Epidemiol.

[43] Zhao L-L, Huang H, Wang Y (2020). Lifestyle factors and long-term survival of gastric cancer patients: a large bidirectional cohort study from China. World J Gastroenterol.

[44] Rota M, Pelucchi C, Bertuccio P (2017). Alcohol consumption and gastric cancer risk—a pooled analysis within the StoP project consortium. Int J Cancer.

[45] Ferronha I, Castro C, Carreira H (2012). Prediagnosis lifestyle exposures and survival of gastric cancer patients: a cohort study from Portugal. Br J Cancer.

[46] Jayalekshmi PA, Hassani S, Nandakumar A (2015). Gastric cancer risk in relation to tobacco use and alcohol drinking in Kerala, India--Karunagappally cohort study. World J Gastroenterol.

[47] Islami F, Goding Sauer A, Miller KD (2018). Proportion and number of cancer cases and deaths attributable to potentially modifiable risk factors in the United States. CA Cancer J Clin.

[48] Agudo A, Bonet C, Travier N (2012). Impact of cigarette smoking on cancer risk in the European prospective investigation into cancer and nutrition study. J Clin Oncol.

[49] Lissowska J, Groves FD, Sobin LH (1999). Family history and risk of stomach cancer in Warsaw, Poland. Eur J Cancer Prev.

[50] Bakir T, Can G, Siviloglu C (2003). Gastric cancer and other organ cancer history in the parents of patients with gastric cancer. Eur J Cancer Prev.

[51] Ikeguchi M, Fukuda K, Oka S (2001). Clinicopathological findings in patients with gastric adenocarcinoma with familial aggregation. Dig Surg.

[52] Yang L, Kartsonaki C, Yao P (2021). The relative and attributable risks of cardia and non-cardia gastric cancer associated with Helicobacter pylori infection in China: a case-cohort study. Lancet Public Health.

[53] Wang F, Sun G, Zou Y (2013). Helicobacter pylori infection predicts favorable outcome in patients with gastric cancer. Curr Oncol.

[54] Varga MG, Wang T, Cai H (2018). Helicobacter pylori blood biomarkers and gastric cancer survival in China. Cancer Epidemiol Biomarkers Prev.

[55] Morishima T, Matsumoto Y, Koeda N (2019). Impact of comorbidities on survival in gastric, colorectal, and lung cancer patients. J Epidemiol.

[56] Wu J, Tian S, Xu J (2023). Association of high-risk comorbidity with overall survival among patients with gastric cancer and its sex-specific differences in China: a retrospective observational cohort study. BMC Cancer.

[57] Osinowo AO, Olajide TO, Balogun OS (2023). Clinicopathological features and treatment outcome of patients with gastric cancer in Lagos: is the outlook getting better?. J West Afr Coll Surg.

[58] Barad AK, Mandal SK, Harsha HS (2014). Gastric cancer – a clinicopathological study in a tertiary care centre of North-eastern India. J Gastrointest Oncol.

[59] Chen YC, Fang WL, Wang RF (2016). Clinicopathological variation of Lauren classification in gastric cancer. Pathol Oncol Res.

[60] Schirren R, Novotny A, Oesterlin C (2021). Significance of Lauren classification in patients undergoing neoadjuvant/perioperative chemotherapy for locally advanced gastric or gastroesophageal junction cancers – analysis from a large single center cohort in Germany. Cancers.

[61] Alshahrani S, Baabbad F, Bahobail M (2020). Survival time in treatment modalities of gastric carcinoma at King Khalid hospital-Jeddah Saudi Arabia: a retrospective cohort study. Mater Socio Med.

[62] Ning FL, Zhang NN, Wang J (2021). Prognostic value of modified Lauren classification in gastric cancer. World J Gastrointest Oncol.

[63] Deng K, Yang L, Hu B (2015). The prognostic significance of pretreatment serum CEA levels in gastric cancer: a meta-analysis including 14651 patients. PLoS One.

[64] Lordick F, Carneiro F, Cascinu S (2022). Gastric cancer: ESMO Clinical Practice Guideline for diagnosis, treatment and follow-up. Ann Oncol.

[65] Al-Batran SE, Homann N, Pauligk C (2019). Perioperative chemotherapy with fluorouracil plus leucovorin, oxaliplatin, and docetaxel versus fluorouracil or capecitabine plus cisplatin and epirubicin for locally advanced, resectable gastric or gastro-oesophageal junction adenocarcinoma (FLOT4): a randomised, phase 2/3 trial. Lancet.

[66] Macdonald JS, Smalley SR, Benedetti J (2001). Chemoradiotherapy after surgery compared with surgery alone for adenocarcinoma of the stomach or gastroesophageal junction. N Engl J Med.

[67] Noh SH, Park SR, Yang HK (2014). Adjuvant capecitabine plus oxaliplatin for gastric cancer after D2 gastrectomy (CLASSIC): 5-year follow-up of an open-label, randomised phase 3 trial. Lancet Oncol.

[68] Ali Z, Mahmoodi M, Mohammad K (2014). Factors affecting the survival of patients with gastric cancer undergone surgery at iran cancer institute: univariate and multivariate analyses. Iran J Public Health.

[69] Basaran H, Koca T, Cerkesli AK (2015). Treatment outcomes and survival study of gastric cancer patients: a retrospective analysis in an endemic region. Asian Pac J Cancer Prev.

[70] Seyedin S, Wang PC, Zhang Q (2014). Benefit of adjuvant chemoradiotherapy for gastric adenocarcinoma: a SEER population analysis. Gastrointest Cancer Res.

[71] Komatsu Y, Hironaka S, Tanizawa Y (2022). Treatment pattern for advanced gastric cancer in Japan and factors associated with sequential treatment: a retrospective administrative claims database study. Adv Ther.

[72] Degu A, Karimi PN, Opanga SA (2023). Predictors of survival outcomes among patients with gastric cancer in a leading tertiary, teaching and referral hospital in Kenya. Cancer Med.

[73] Alshehri A, Alanezi H, Kim BS (2020). Prognosis factors of advanced gastric cancer according to sex and age. World J Clin Cases.

[74] Song YX, Huang XZ, Gao P (2015). Clinicopathologic and prognostic value of serum carbohydrate antigen 19–9 in gastric cancer: a meta-analysis. Dis Mark.

[75] Greene F, Page D, Fleming I (2002). Cancer Staging Manual.

